# Real-World Analysis of Outcomes and Economic Burden in Patients with Chronic Kidney Disease with and without Secondary Hyperparathyroidism among a Sample of the Italian Population

**DOI:** 10.3390/nu15020336

**Published:** 2023-01-10

**Authors:** Simona Barbuto, Valentina Perrone, Chiara Veronesi, Melania Dovizio, Fulvia Zappulo, Daniele Vetrano, Sandro Giannini, Maria Fusaro, Domenica Daniela Ancona, Antonietta Barbieri, Fulvio Ferrante, Fabio Lena, Stefano Palcic, Davide Re, Francesca Vittoria Rizzi, Paolo Cogliati, Marco Soro, Luca Degli Esposti, Giuseppe Cianciolo

**Affiliations:** 1 Nephrology, Dialysis and Kidney Transplant Unit, IRCCS Azienda Ospedaliero-Universitaria di Bologna, 40138 Bologna, Italy; 2 CliCon S.r.l., Società Benefit, Health, Economics & Outcomes Research, 40137 Bologna, Italy; 3 Clinica Medica 1, Department of Medicine, University of Padova, 35128 Padova, Italy; 4 National Research Council (CNR), Institute of Clinical Physiology (IFC), 56124 Pisa, Italy; 5 Department of Medicine, University of Padova, 35128 Padova, Italy; 6 Pharmaceutical Department, ASL BAT (Barletta Andria Trani), 76123 Andria, Italy; 7 Direttore SC Farmaceutica Territoriale, ASL VC, 13100 Vercelli, Italy; 8 UOC Farmacia, Ufficio di Farmacovigilanza, ASL Frosinone, 03100 Frosinone, Italy; 9 U.O.C. Politiche del Farmaco, USL Toscana Sud Est, 58100 Grosseto, Italy; 10 SC Farmacia Ospedaliera e Territoriale—Area Giuliana, Azienda Sanitaria Universitaria Integrata Giuliano-Isontina (ASUGI), 34128 Trieste, Italy; 11 Servizio Farmaceutico Territoriale, ASL Teramo, 64100 Teramo, Italy; 12 CSL Vifor, 00142 Rome, Italy

**Keywords:** chronic kidney disease, secondary hyperparathyroidism, real-world evidence, CKD-MBD (Chronic Kidney Disease-Mineral Bone Disorder), native vitamin D, active vitamin D

## Abstract

This real-world analysis evaluated the clinical and economic burden of non-dialysis-dependent CKD patients with and without secondary hyperparathyroidism (sHPT) in Italy. An observational retrospective study was conducted using administrative databases containing a pool of healthcare entities covering 2.45 million health-assisted individuals. Adult patients with hospitalization discharge diagnoses for CKD stages 3, 4, and 5 were included from 1 January 2012 to 31 March 2015 and stratified using the presence/absence of sHPT. Of the 5710 patients, 3119 were CKD-only (62%) and 1915 were CKD + sHPT (38%). The groups were balanced using Propensity Score Matching (PSM). Kaplan-Meier curves revealed that progression to dialysis and cumulative mortality had a higher incidence in the CKD + sHPT versus CKD-only group in CKD stage 3 patients and the overall population. The total direct healthcare costs/patient at one-year follow-up were significantly higher in CKD + sHPT versus CKD-only patients (EUR 8593 vs. EUR 5671, *p* < 0.001), mostly burdened by expenses for drugs (EUR 2250 vs. EUR 1537, *p* < 0.001), hospitalizations (EUR 4628 vs. EUR 3479, *p* < 0.001), and outpatient services (EUR 1715 vs. EUR 654, *p* < 0.001). These findings suggest that sHPT, even at an early CKD stage, results in faster progression to dialysis, increased mortality, and higher healthcare expenditures, thus indicating that timely intervention can ameliorate the management of CKD patients affected by sHPT.

## 1. Introduction

Chronic kidney disease (CKD) is a serious global public health emergency, with a rising burden during the last decades, along with the parallel increase in diabetes, cardiovascular disease, and an ageing population [1]. The current prevalence is estimated to be around 15% in the adult population of the United States [2] and 13.4% globally [3], while in Italy, a lower prevalence, on average of 7%, has been reported [4]. The progression of CKD is associated with an increased risk of death, cardiovascular events, and hospitalization [5,6]. Among the underlying causes, mineral metabolism alterations have been implicated, including derangement of calcium-phosphate homeostasis [7], which leads to secondary hyperparathyroidism (sHPT) characterized by elevated parathyroid hormone (PTH) levels and parathyroid hyperplasia [8,9,10,11,12]. In 2005, the KDIGO (Kidney Disease Improving Global Outcomes) guidelines coined the definition of CKD-MBD (Chronic Kidney Disease-Mineral Bone Disorder) involving a broad spectrum of bone and cardiovascular diseases, mostly driven by impaired mineral metabolism and sHPT, occurring in approximately 40 to 80% of CKD patients in stages 3 or 4 [13]. High serum phosphate stimulates the release of fibroblast growth hormone 23 (FGF23) from the bone which, in turn, increases PTH levels by inhibiting the activation of 25-hydroxyivitamin D (25(OH)-D) in 1,25-dihydroxyvitamin D (1,25 (OH)2-D) through the suppression of renal 1α-hydroxylase expression [14,15]. Failure to correct modifiable factors such as vitamin D deficiency and hyperphosphatemia induces a maladaptive response characterized by initial polyclonal hyperplasia of the parathyroid cells, ultimately triggering sHPT onset and progression [16]. These modifications are associated with a progressive reduction in the expression of receptors for calcium (CaSR), calcitriol (VDR), and FGF-23 (FGFR1-Klotho), leading to a reduced sensitivity in the parathyroid cells to the inhibitory effect of calcium, calcitriol, and FGF-23 and a worsening of hyperparathyroidism [17,18]. The most recent KDIGO guidelines (2017) for the management of CKD-MBD state the importance to monitor serum levels of calcium, phosphate, and PTH at the beginning of CKD stage G3a, and to evaluate their trends over time, as well as suggesting measuring levels of 25(OH)D to diagnose vitamin D deficiency. Nonetheless, KDIGO guidelines do not specify an optimal PTH value in patients with CKD stage G3a-G5 but suggest interventions to keep PTH between two and nine times the normal values in stage G5d [19]. In addition, the KDOQI (Kidney Disease Outcome Quality Initiative) working group recommend PTH values to be maintained between 150 and 300 pg/mL in patients with CKD stage G5d [13].

Therapies currently prescribed for sHPT management include native vitamin D (NVD, cholecalciferol, ergocalciferol), vitamin D prohormone (extended-release calcifediol), active vitamin D (AVD) and analogs (calcitriol, paricalcitol), and calcimimetics (only for G5d). In adult patients with CKD stages G3a-G5 who are not on dialysis, AVD and analogs are not recommended for routine use, while it has been suggested to reserve their utilization for patients with CKD stages G4-G5 with severe and progressive sHPT and to modulate their administration on the basis of serial measurements of phosphate, calcium, and PTH levels [19,20,21]. On the other hand, the persistently elevated PTH values in non-dialysis patients have been implicated in worsening CKD, cardiovascular events [22,23,24,25], and massive use of sHPT therapies 9–12 months after starting dialysis to correct the extremely high PTH values [26].

The presence of sHPT in CKD and ESRD (end-stage renal disease) implies a heavy economic burden related to high incremental healthcare costs, mostly driven by the costs of therapies for sHPT and for the resources used to treat sHPT-related clinical events [27,28,29]. However, poor evidence is currently available on the sHPT clinical and economic outcomes for non-dialysis dependent CKD (ND-CKD) patients in the Italian clinical routine setting. 

The aim of the present analysis was to evaluate the epidemiology and clinical and economic burden of CKD patients with and without sHPT, in a real-world Italian setting, and to estimate healthcare resource consumption covered by the Italian National Health System (INHS).

## 2. Materials and Methods

### 2.1. Data Source

An observational retrospective study based on administrative databases containing a sample of Italian Entities covering approximately 2.45 million of health-assisted individuals was performed. For the current analysis, the Italian Local Health Units database was selected because of its geographical distribution, data completeness, and high-quality linked datasets. Within the administrative flows, the anonymous univocal numeric code assigned to each patient allowed the electronic linkage of all the patients’ records across the databases. Specifically, the data linkage was performed among the following databases: the beneficiaries database that contains patient demographic data such as gender, year of birth, and date of death; the pharmaceuticals database that contains data on prescriptions as Anatomical Therapeutic Chemical (ATC) code, marketing authorization code, number of packages, number of units per package, unit cost per package, and prescription date; the hospitalization database that includes all hospitalization data such as admission and discharge date, type of hospitalization, primary and secondary (up to five) discharge diagnosis codes classified according to the International Classification of Diseases, Ninth Revision, Clinical Modification (ICD-9-CM), procedure ICD-9-CM codes (primary and up to five secondary procedure diagnosis), Diagnosis Related Group (DRG), and DRG related charge; the outpatient specialist services database, which contains date of prescription, type, description activity of diagnostic tests, and visits for patients in analysis and laboratory test or specialist visit charge; the payment waivers database that includes the date and code of exemption that allow the citizen to avoid the economic copayment for services/treatments in the presence of certain diseases (payment waiver code). The anonymous univocal numeric code ensured total compliance with the European General Data Protection Regulation (GDPR) (2016/679). The integration of administrative datasets allowed the representation of the patient’s entire clinical history and not just individual prescriptions. The analyses were conducted using exclusively anonymous data in full compliance with privacy regulations. The results are exclusively presented in aggregated form and are not attributable to a single institution, department, doctor, individual, or individual prescribing behaviors. The analysis was conducted in accordance with the Helsinki Declaration and in full compliance with current legislation for retrospective studies. Based on the Data Privacy Guarantor Authority (General Authorization for personal data treatment for scientific research purposes—n.9/2014), informed consent was not required, as its collection would be impossible for organizational reasons. According to Italian law on the conduction of observational analyses [30], the ethics committee of each participating entity was notified and approved the analysis.

### 2.2. Study Population and Cohorts

All patients were enrolled in the study if they presented at least one hospitalization with primary or secondary discharge diagnosis for CKD (any stage, 3–5) between 1 January 2012 and 31 March 2015 (enrolment period). The diagnosis was identified using ICD-9-CM codes: 585.3-585.4-585.5. The date of first diagnosis during this period was defined as the “CKD-index date”. Among patients identified, the presence of an sHPT diagnosis was ascertained during the first two years after the CKD-index date by at least one prescription of paricalcitol (ATC code: H05BX02) or calcitriol (ATC code: A11CC04), or a value of PTH ≥ 85 pg/mL, or at least one hospitalization with a discharge diagnosis of sHPT (ICD-9-CM: 252.0), or a payment waiver code for sHPT (026.252.0). Patients were excluded if they were under 18 years of age at the index date or if they had one of the following diagnoses at the CKD-index date or prior to their inclusion: dialysis, cancer, HIV, HCV, or cirrhosis. The included patients were assigned to one of two mutually exclusive cohorts: (1) the “CKD-only” cohort that included patients without sHPT diagnosis for whom the index date corresponded to the CKD-index date or (2) the “CKD + sHPT” cohort including CKD patients with sHPT diagnosis for whom the index date was set at the first match with the sHPT criteria. The patients were characterized during all data availability periods before the index date (characterization period) and the follow-up from the index date until 31 July 2020 (follow-up period).

### 2.3. Baseline Demographic and Clinical Patient Characteristics

Demographic variables such as age and gender were collected at the index date, while the presence of the following comorbidities was investigated during the characterization period using, as a proxy for diagnosis, hospitalization discharge diagnosis and/or specific treatments: cardiovascular disease-related comorbidities, i.e., hypertension (identified by at least 1 hospitalization with a discharge diagnosis for essential hypertension (ICD-9-CM codes: 401) or at least 1 prescription of antihypertensive drugs (ATC codes: C02, C03; C07; C08; C09)); diabetes mellitus (identified by at least 1 hospitalization with a discharge diagnosis for diabetes mellitus (ICD-9-CM code: 250) or at least 1 prescription of antidiabetic drugs (ATC code: A10)); dyslipidemia (identified by at least 1 hospitalization with a discharge diagnosis for disorders of lipid metabolism (ICD-9-CM code: 272) or at least 1 prescription of lipid modifying agents (ATC code: C10)); cardiovascular disease (identified by at least 1 hospitalization with a discharge diagnosis for ischemic heart disease (ICD-9-CM codes: 410, 411, 413, 414), cerebrovascular disease (ICD-9-CM codes: 430, 431, 432, 433, 434, 435, 436, 437, 438), atherosclerosis (ICD-9-CM code: 440) and other peripheral vascular disease (ICD-9-CM codes: 443)); or osteoporosis (identified by at least 1 hospitalization with a discharge diagnosis for osteoporosis (ICD-9-CM code: 733.0) or at least 1 prescription of osteoporotic drugs (ATC codes M05BA, M05BB, M05BX, H05AA, H05BA, G03XC)) [31,32]. During the characterization period, the prescription of diuretics (ATC code: C03), which are often used for patients with CKD or antiplatelets (ATC code: B01AC), as a proxy of cardiovascular comorbidities, were evaluated in view of the known relationship of these drugs with PTH levels. Specifically, AVD (i.e., calcitriol, ATC code A11CC04 or paricalcitol ATC code: H05BX02) and NVD (i.e., ergocalciferol, ATC code A11CC01 or cholecalciferol, ATC code A11CC05, or calcifediol, ATC code A11CC06) were evaluated. During the follow-up period, the occurrence of death and CKD progression to dialysis were assessed.

### 2.4. Outcome Evaluation

During the follow-up, the occurrence of mortality and the initiation to dialysis were evaluated with Kaplan-Meier curves, which were used to report the probability of surviving to the outcome events. The incident rate/1000-person years was also evaluated.

### 2.5. Healthcare Resource Consumptions and Costs

Overall healthcare resource consumption was evaluated during the follow-up period (by excluding the index-date) in terms of drugs prescribed, hospitalizations, and outpatient services. The mean annual all-cause direct healthcare costs per patient based on resource consumption was evaluated during the follow-up. The healthcare cost analysis was performed from the perspective of the INHS, with costs reported in Euros (EUR). Drug costs were evaluated using the INHS purchase price. Hospitalization costs were determined using DRG tariffs, which represent the reimbursement levels from the INHS healthcare providers. The costs of specialist and laboratory tests were defined according to the tariffs applied in each region.

### 2.6. Statistical Analysis

Continuous variables are reported as mean ± standard deviation (SD) and categorical variables are expressed as numbers and percentages. A *p*-value < 0.05 was considered statistically significant. Given the study’s observational nature, the Propensity Score Matching (PSM) methodology was used to abate potential unbalances both in baseline characteristics among the cohorts (CKD-only versus CKD + sHPT patients, and CKD + sHPT patients untreated versus patients treated with AVD/NVD). Patients were matched on quintiles of propensity score calculated using a logistic regression model, which included the following variables: age, sex, presence of diabetes, cardiovascular disease/treatments, osteoporosis, and CKD stage. To maintain a maximum number of patients, a 1:1 matching algorithm was used. All analyses were performed using Stata SE version 17.0 (StataCorp, College Station, TX, USA).

## 3. Results

**Figure 1:**
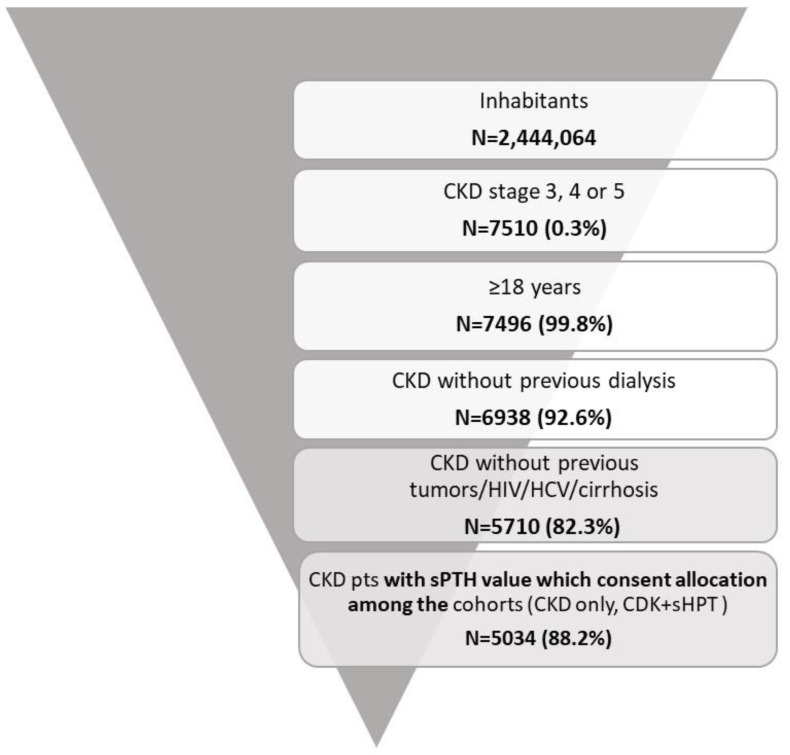
Flow-chart for the definition of cohorts included in the analysis.

Among the sample population, using the application of inclusion and exclusion criteria, 5034 CKD patients were included and allocated into the two cohorts: 3119 patients were allocated into the CKD-only cohort (62%) and 1915 patients were allocated into the CKD + sHPT cohort (38%) (Figure 1).

**Figure 2:**
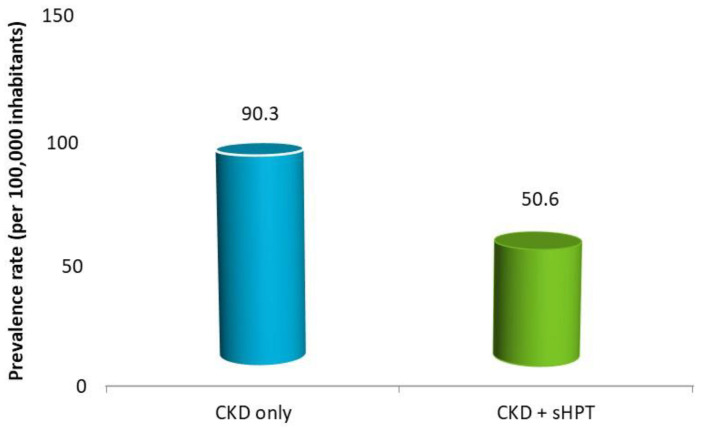
Estimated prevalence in patients with a diagnosis of CKD without (CKD-only) and with sHPT diagnosis (CKD + sHPT).

As reported in Figure 2, the estimated prevalence of CKD-only was 90.3 cases per 100,000 (0.1%) health-assisted individuals; that of CKD + sHPT was 50.6 cases/100,000 individuals (0.05%).

After the PSM methodology, 1590 CKD-only and CKD + sHPT patients were included (Table 1).

The mean age was 78 years, and 56% were males. The patients were adjusted with respect to the baseline variables (age, sex, diabetes, cardiovascular disease/treatments, osteoporosis, and CKD stage). The two cohorts were characterized using the high frequency of cardiovascular disease and related treatments (98% of CKD-only and 98.8% of CKD + sHPT patients), and the use of osteoporosis medications accounted for 10% of the CKD-only and 10.4% of CKD + sHPT patients; 60.9% and 59.0% of CKD-only and CKD + sHPT patients, respectively, were included with CKD stage 3.

**Figure 3:**
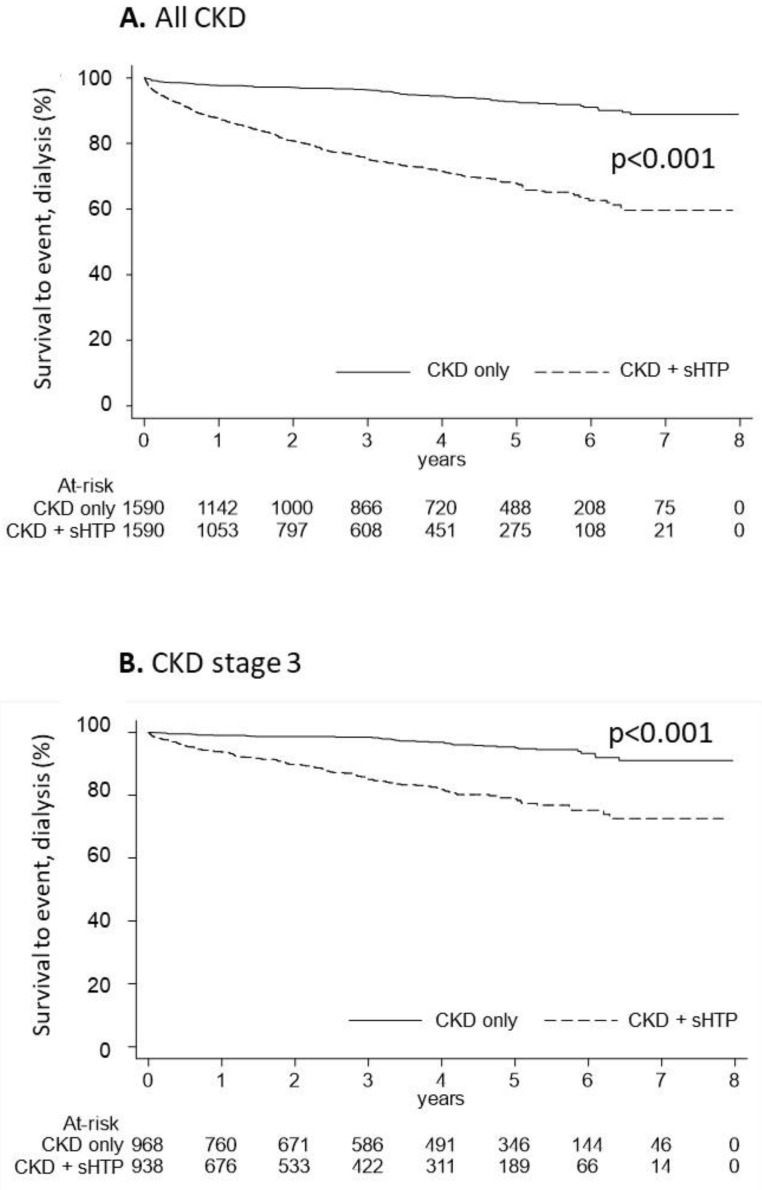
Kaplan-Meier curves for the CKD + sHPT vs. CKD only groups showing survival to the event (dialysis) in all CKD patients (**A**) and in those with CKD stage 3 (**B**).

Progression to dialysis and mortality events were evaluated in overall patients as reported in Figure 3. During the follow-up, the CKD progression to dialysis was significantly more represented in the CKD + sHPT versus CKD patients (incidence rate/100,000: 90.66 in CKD + sHPT and 15.95 in CKD patients, *p* < 0.001) (Figure 3A). This trend was evident in patients at stage 3 (incidence rate/100,000: 51.35 in CKD + sHPT and 10.32 in CKD patients) (Figure 3B), suggesting a possible link between the time of exposure to high PTH and clinical outcomes. 

**Figure 4:**
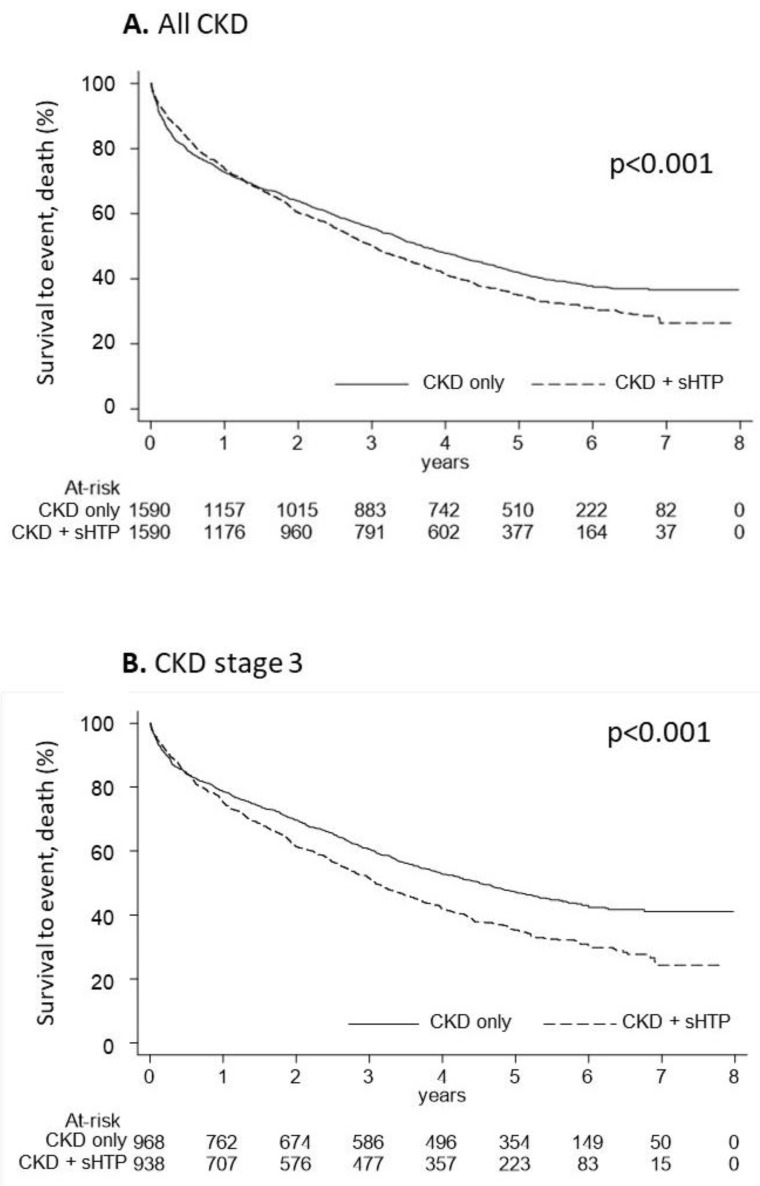
Kaplan-Meier curves for the CKD + sHPT vs. CKD-only groups showing overall survival in all CKD patients (**A**) and in those with CKD stage 3 (**B**).

The Kaplan-Meier curves used to estimate cumulative mortality showed that overall survival was significantly lower (*p* < 0.001) in the CKD + sHPT versus CKD-only group (incidence rate: 217.98 in CKD + sHPT and 180.04 in CKD patients) (Figure 4A). This difference was also evident for patients at stage 3 (incidence rate: 215.61 in CKD + sHPT and 151.69 in CKD patients) (Figure 4B). 

**Figure 5:**
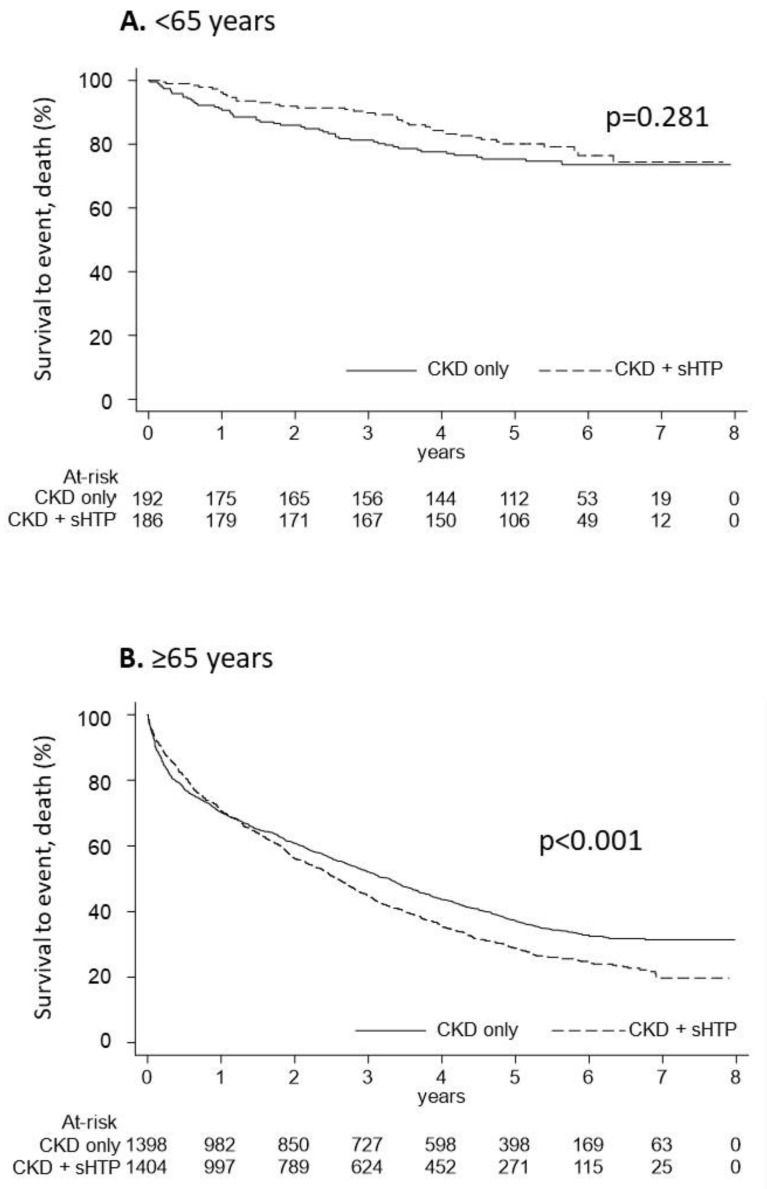
Kaplan-Meier curves for the CKD + sHPT vs. CKD only groups showing overall survival in CKD patients aged < 65 years (**A**) and in those aged ≥ 65 years (**B**).

In Figure 5, the overall survival of CKD and CKD + sHPT patients under and over 65 years old is reported. In patients under 65 years old, the Kaplan-Meier curves showed that overall survival was comparable (*p* = 0.281) among the CKD + sHPT versus CKD-only group (incidence rate: 42.96 in CKD + sHPT and 53.34 in CKD patients) (Figure 5A). In patients ≥65 years old, the Kaplan-Meier curves showed that overall survival was significantly lower (*p* < 0.001) in the CKD + sHPT versus CKD-only group (incidence rate: 259.53 in CKD + sHPT and 206.44 in CKD patients) (Figure 5B). 

Regardless of the vitamin D treatment (NVD, AVD, and analogues), CKD + sHPT patients were subgrouped into those treated or untreated if they presented or not at least one prescription of AVDs and/or NVDs during all available periods. The treated and untreated cohorts were balanced using PSM, and their baseline characteristics are reported in Table 2. 

**Figure 6:**
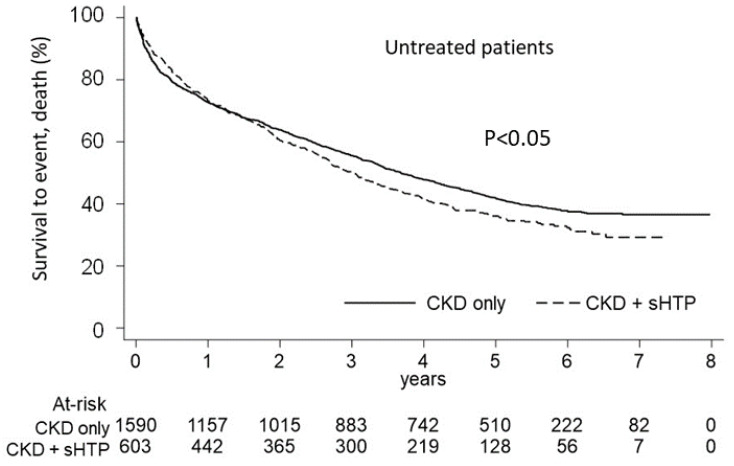
Kaplan-Meier curves for the CKD + sHPT vs. CKD-only groups showing overall survival in untreated patients.

As Figure 6 shows, in the untreated patients, the Kaplan-Meier curves for cumulative mortality revealed a lower survival in the CKD + sHPT versus CKD-only group (*p* < 0.05), with an incidence death rate higher in the CKD + sHPT vs. CKD-only patients (215.96 vs. 180.04, respectively).

Using a comparison of the incidence rate of mortality between the treated and untreated CKD + sHPT patients, it was shown that treated patients were characterized by a significantly lower mortality incidence (192.62/1000-person years) with respect to untreated patients (206.03/1000-person years, *p* < 0.001).

**Figure 7:**
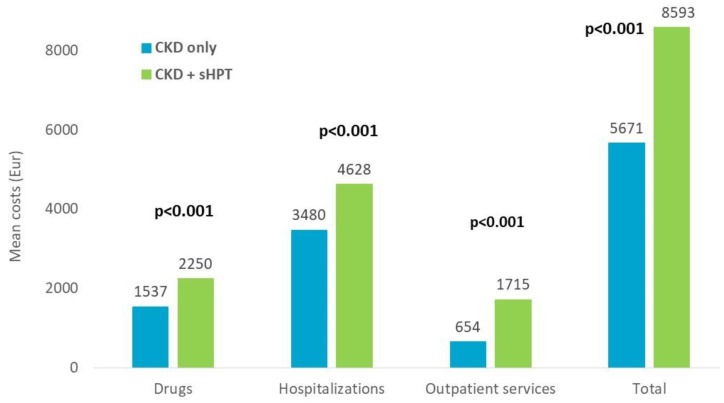
Direct total healthcare costs during the first year of follow up, index-date excluded, post PSM.

The estimation of overall direct healthcare costs during the one-year follow-up (index-date excluded) is reported in Figure 7. The overall mean cost/patient was significantly higher in the CKD + sHPT versus CKD patients without sHPT (EUR 8593 vs. EUR 5671, *p* < 0.001), and this phenomenon derived from higher expenditures related to drugs (EUR 2250 vs. EUR 1537, *p* < 0.001), hospitalizations (EUR 4628 vs. EUR 3479, *p* < 0.001), and outpatient services (EUR 1715 vs. EUR 654, *p* < 0.001) for the management of CKD + sHPT patients with respect to those without sHPT (Figure 7).

## 4. Discussion

The present real-world analysis of data, deriving from the clinical management of CKD patients with and without sHPT in an Italian setting, evaluated the epidemiology of CKD with and without sHPT (CKD-sHPT and CKD-only), the occurrence of CKD disease progression and mortality in these patients, and the estimation of the economic burden from their clinical management.

In this analysis, the prevalence of CKD (stage 3–5) was 0.3%, and for specific CKD phenotypes with and without sHPT, the prevalence was reported as 0.1% and 0.05%, respectively. In Italy, a few different studies have been conducted to estimate the prevalence of CKD. The INCIPE (Initiative on Nephropathy, of relevance to public health, which is Chronic, possibly in its Initial stages, and carries a Potential risk of major clinical End-points) study analyzed the estimated glomerular filtration rate (eGFR) and albumin to creatinine ratio in a cohort of 6200 individuals (randomly selected among the residents in the Northeast) aged above 40 years and found a prevalence of CKD (considering all stages) of 13.2% [33]. Similarly, in the VIP (Valle dell’Irno Prevenzione) project, conducted in an area of Southern Italy, the prevalence of CKD (stage 3–5) was estimated at around 5.9% [34]. The CARHES (CArdiovascular risk in Renal Patients of the Italian Health Examination Survey) study analyzed the general population between 35 and 79 years old and found a prevalence of CKD stages 3–5 of 2.89% [4]. Likewise, the MATISS (Malattia ATerosclerotica Istituto Superiore di Sanità) study on a cohort of individuals aged 35 to 74 (extracted from the electoral lists of a geographical area) found a prevalence of CKD stage 3–5, adjusted for sex and age, of 1% [35]. It should be noted that the INCIPE and VIP studies estimated eGFR with the Cockcroft-Gault formula [36], and in the MATISS and CARHES studies, the CKD-EPI (Chronic Kidney Disease Epidemiology Collaboration) equation was used [37]. In the present study, we found even lower values for the CKD prevalence, primarily explained by the discrimination of ND-CKD patients with a single diagnosis from those with a co-diagnosis of sHPT. Another explanation could be that in the present retrospective analysis, CKD was identified using hospitalization discharge diagnosis, and instead, the other studies analyzed data from the general population. It should be noted that the prevalence of CKD stages 3–5 found in the present analysis as well as in other Italian studies appears to be among the lowest across Europe [38] and significantly lower compared to the US data [2]. There are several reasons for such epidemiological differences in the various geographical areas, such as feasibly related to ethnicity [39,40] and dietary habits (i.e., protein intake known to affect serum creatinine and thus eGFR) [41], public health policies to control renal diseases using primary and secondary prevention measures [42], inconsistency in the assays used for serum creatinine [43] and albuminuria measurements [44], and heterogeneity in the study populations or observation periods [38,45,46].

After PSM, an adjustment of the study cohort variables was obtained, and the occurrence of outcomes and the analysis of healthcare costs were evaluated in covariate-adjusted cohorts. 

The probability of CKD progression to the dialysis event was significantly higher in the CKD + sHPT patients concerning CKD-only with an incidence rate of 90.66/1000 person-year as the probability to survive to the event was significantly lower in CKD-sHPT patients (*p* < 0.001) compared to the other group. This phenomenon was also evident in patients with early-stage CKD, in fact, CKD stage 3 patients had an incidence rate of 51.35/1000-person years compared to 10.32/1000-person years in the CKD-only group. Several studies have obtained similar results. For example, Xu et al conducted an observational study on 2556 patients with CKD stage 1–5 and showed that the progression of CKD was associated with an increase in the incidence of sHPT (57 cases/1000 person-years in CKD stage G3 versus 230 cases/1000 person-years in stage G5) and at the same time, the onset of sHPT was associated, after a multivariable adjustment, with a 5 times greater risk of CKD progression [24]. It is worthy to note, although a supposition only, that the time-dependent exposure and its effect on clinical outcomes might suggest that an early PTH control can trigger a delayed initiation of dialysis, with clear consequences for patients and healthcare systems in terms of health-related quality of life and budget. Similarly, Bozic et al, in an observational study on 2445 patients from the NEFRONA (National Observatory of Atherosclerosis in Nephrology) cohort, showed that after 2 years of follow-up, the presence of sHPT at baseline was independently associated with the progression of CKD [22]. The mechanism behind this relationship is unclear; it has been suggested that the increase in FGF-23, following the increase in PTH and hyperphosphatemia, may induce a worsening of renal function and a progression towards dialysis. A study using mouse models with cardio-renal syndrome post-myocardial infarction showed that elevated FGF-23 levels were associated with a profibrotic effect on renal and cardiac levels, through the binding between FGF-23 and its receptor FGFR4 in the kidney that induces β-catenin signaling pathway activation; however, this effect was reduced if an FGFR antagonist was administered [47]. This data was confirmed in a prospective study by Seiler et al, who used a cohort of 312 patients with stage 2–4 CKD after 2.2 follow-up years and found that elevated FGF-23 levels were an independent predictor of death or dialysis (HR 2.49; 95% CI, 1.12–5.54; *p* = 0.025) [48]. Furthermore, it is important to consider that FGF-23 reduces the expression of a-Klotho in the kidney and parathyroid glands in a dose-dependent manner; therefore, high levels of FGF-23 in sHPT induce a further reduction of a-Klotho, which predisposes renal damage. In this regard, Lindberg et al generated a mouse model with a-Klotho deleted throughout the nephron and demonstrated how histologically there was an increase in cell density, loss of differentiation between proximal and distal convoluted tubules, and loss of cuboid epithelium of the Bowman’s capsule associated with interstitial fibrosis and widespread nephrocalcinosis. In addition, there were ectopic calcifications at renal, pulmonary, and arterial levels [49]. Similarly, Kim et al completed an observational study with 243 CKD patients divided into two groups based on circulating levels of a-Klotho and showed that 35.2% of the group that had lower levels reached the primary composite outcome (doubling of baseline serum creatinine concentration, end-stage renal disease, or death) compared to 15.7% of the group with high a-klotho levels (HR, 2.03; 95% CI, 1.07–3.85; *p* = 0.03). Furthermore, end-stage renal disease occurred in 17.2% of the patients with lower a-klotho compared to 7.4% of the other group (*p* < 0.001) [50].

An interesting finding obtained from this analysis was that of mortality, which has already been shown in the literature. Patients in the CKD + sHPT group had an incidence rate of death of 217.98/1000-person year compared to 180.04/1000-person year in the CKD-only group; therefore, survival was significantly reduced in the first group (*p* < 0.001). The trend was more evident in patients older than 65 years and mainly, after stratification based on the degree of CKD, in the CKD + sHPT stage 3 patients that had a higher incidence rate (215.61 vs. 151.69/1000-person years) and reduced survival (*p* < 0.001). We assumed that the data were less significant in more advanced stages due to greater comorbidities and more compromised renal function. Several studies have confirmed this finding. Geng et al analyzed data from 5108 (mean age 68 + 17 years) patients from Marshfield Clinic Health System electronic health records and found that PTH was an independent predictor of fractures, vascular events, and death [51]. Similarly, the study by Xu mentioned above found that elevated PTH values increased the risk of death by 1.3 times (95% confidence interval 1.1–1.8) [24]. Shardlow et al analyzed a population of 1741 patients with stage 3 CKD for 5 years and recorded death in 18.4% of participants (34.9% from cardiovascular causes), and they also found that the presence of sHPT (high PTH values and vitamin D deficiency) was significantly associated with all causes of death [52]. The correlation between sHPT and mortality can be explained by several factors: a reduction of vitamin D and an increase of PTH and FGF-23. These contribute, according to various pathogenetic pathways, to favoring vascular calcifications, left ventricular hypertrophy, and the risk of arrhythmias and, therefore, increase mortality, particularly from cardiovascular events.

In our cohorts in stage 3, the CKD-sHPT group had a hyperphosphatemia incidence rate of 139.25/1000-person year compared to the CKD-only group (37.36/1000-person year), and this result increased as CKD progressed. In fact, in the CKD-sHPT stages 4 and 5 groups, it was 211.16 and 574.59/1000-person year, respectively. However, in the CKD-only group, the values were 59.46 and 45.4/1000-person year, respectively. Furthermore, the probability to survive to the hyperphosphatemia events in the CKD + sHPT patients was significantly lower with respect to CKD-only in all stages (*p* < 0.001). This is an important finding as it is known that hyperphosphatemia is among the non-traditional factors that increase the risk of cardiovascular events in patients with CKD. As demonstrated using a meta-analysis, an increase in phosphate correlates with an increase in cardiovascular risk (aggregate hazard ratio 1.20 per mg/dL increase, 95% CI 1.08–1.33, *p* < 0.005). In fact, hyperphosphatemia induces some mechanisms of endothelial dysfunction and oxidative stress and stimulates the switch of vascular smooth muscle cells in an osteochondrogenic phenotype in a dose-dependent manner, which results in an increase in vascular and valve calcifications. In 2009, Adeney et al completed a prospective multicenter study on 439 patients with CKD and without cardiovascular disease and showed that an increase of 1 mg/dl of phosphate in the blood was associated with an increase in coronary, thoracic aorta, and aortic and mitral valves calcifications (21%, 33%, 25 and 62%, respectively) [53]. Similarly, Bundy et al. analyzed 1123 CKD patients enrolled in the CRIC Study and found that after 3.3 years of follow-up, 25.9% of patients had developed (de novo) and 18% had progressed in measured coronary calcifications with the Agatson score; one of the most important risk factors was hyperphosphatemia [54].

Finally, an important result obtained from the analysis concerns the impact of sHPT treatment in patients with CKD. After PSM, 690 treated and untreated patients with an average age of 76 years were included in the cohort. Patients were matched based on age, sex, diabetes, cardiovascular disease/treatments, osteoporosis, and CKD stage variables, which were comparable in the two cohorts. Among the treated patients, those who underwent therapy with nutritional or AVD or a combination of the two were considered, with the mean follow-up being 3.1 (untreated) and 3.3 (treated) years. The incidence rate of death was significantly lower in the treated than in untreated patients (192.62 vs. 206.03/ 1000-person years, respectively, *p* < 0.001) and, similarly, the incidence rate for a dialysis event was significantly lower in the treated patients (94.89 vs. 110.59/1000-person years, *p* < 0.001). On the other hand, no statistically significant differences were found regarding cardiovascular events and fractures; however, the incidence rate of hypercalcemia in the treated patients compared to untreated patients was significant (47.95 vs. 37.03/1000-person years respectively, *p* < 0.001). As mentioned above, the treatment of sHPT has been much discussed to date. Regarding vitamin D therapy, the 2003 KDOQI guidelines suggest starting therapy with AVD in the CKD stages G3-G4 when the levels of 25(OH)-vitamin D are above 30 ng/mL (75 nmol/L) and the PTH values are higher than the target [13]. The KDIGO guidelines suggest instead considering the use of calcitriol and vitamin D analogues in the CKD stages G4-G5 with severe and progressive sHPT [19]. 

Vitamin D plays a fundamental role in mineral homeostasis in connection with PTH and FGF-23. The increase in FGF-23 entails the reduction of both 25(OH)-D and 1,25(OH)_2_-D by activating the vitamin D catabolic feedback mechanism involving 24-hydroxylase. Low levels of 1,25 (OH)_2_-D promote the progression of secondary hyperparathyroidism by means of multiple pathways, including decreased intestinal absorption of calcium that further stimulates PTH secretion. Vitamin D insufficiency is highly prevalent among patients with CKD, being more common than in the general population and affecting 71–84% of patients with CKD stage 3–4, respectively (insufficiency defined as a level of 25 (OH)-D ≤ 30 ng/mL). It is currently unknown whether intact PTH (iPTH) normalization is an appropriate target for both vitamin D deficiency repletion therapy and AVD and analogues treatment. However, it is known that the optimal level of iPTH in patients with stage 3 or 4 chronic renal failure remains undefined. The KDIGO guidelines suggest that modest increases in PTH represent an appropriate adaptive response to decline in renal function and that overly aggressive use of AVD and analogues increases the risk of adynamic bone disease [19].

Several studies have evaluated the impact of NVD therapy on the progression of CKD and sHPT. A randomized, double-blind, placebo-controlled study conducted on 95 subjects by Westerberg et al showed that after 12 weeks, high-dose cholecalciferol therapy (8000 IU/day) improved PTH values in the treated group compared to placebo (the mean change of PTH in the treated group was −0.76 ± 3 pmol/L compared to 1.6 ± 5 pmol/L in the untreated group) [55]. Similar results were obtained by Alvarez in a double-blind, randomized, placebo-controlled trial in which 46 subjects with early CKD (stages 2–3) were given cholecalciferol (50,000 IU/week for 12 weeks followed by 50,000 IU/every other week for 40 weeks) with a significant reduction in PTH values from baseline (baseline 89.1 ± 49.3 to 70.1 ± 24.8 pg/mL; *p* = 0.01) [56]. A recent meta-analysis of 14 studies showed that the reduction in PTH in relation to cholecalciferol therapy is especially significant when compared to the placebo/no treatment groups (pooled difference in PTH: −49.7 pg/mL, 95%CI −70.2 to −29.3) and does not report significant changes in phosphate or FGF-23 values [57].

Overall, based on the available studies, it appears that nutritional vitamin D may be effective in preventing further increases in PTH but less effective in reducing PTH in patients with high or increasing levels. Moreover, active supplementation correlates with an increased risk of hypercalcemia and hyperphosphatemia. In addition, AVDs are also associated with an increased risk of hypercalcemia and the risk of accelerated vascular calcification. Some studies have assumed that vitamin D therapy and maintenance of adequate vitamin D values could reduce the progression of CKD, as suggested by our analysis. Ferandez-Juarez et al demonstrated that circulating 25(OH)-D values were associated with a reduction in the risk of death and progression of CKD in 103 patients with diabetic nephropathy [58] who were part of the multicenter randomized controlled PRONEDI (Progression of Type 2 Diabetic Nephropathy) trial [59]. Among the participants, 51.5% had values of 25(OH)-D < 15 ng/mL, and this was associated with the primary composite endpoint of a 50% increase in serum creatinine concentration, ESRD, or death (hazard ratio, 2.88; 95%CI: 1.84 to 7.67; *p* = 0.04) [58]. Similarly, Molina et al conducted a single-center, controlled, open-label study of 101 patients and demonstrated a reduction in the urinary albumin-to-creatinine ratio (uACR) from 284 to 167 mg/g at 6 months (*p* < 0.001) in the group treated with cholecalciferol 666 IU/day as well as a reduction in PTH [60].

Calcifediol (25-hydroxyvitamin D_3_) is the long-lasting circulating form of vitamin D prohormone. A promising future alternative approach could be represented by extended-release calcifediol, which has demonstrated its efficacy in two multicenter, randomized, double-blind, placebo-controlled studies in 429 patients with CKD stage G3-G4 in which 30 mg of extended-release calcifediol was used. After 12 weeks, in 95% per-protocol subjects, the 25(OH)_2_D values were normalized and there was a reduction in PTH values of up to 50% at 52 weeks. No changes in calcium, phosphorus, or FGF-23 were reported [61].

Our analysis further reiterates the need to define an effective and timely treatment of sHPT, possibly tailored to the severity of the metabolic framework, aimed at preventing the progressive morpho-functional adaptations that take place in severe and progressive sHPT and which lead to parathyroid gland autonomy. Moreover, the present analysis showed that the management of CKD-sHPT patients was associated with higher direct healthcare costs covered by the INHS, thus suggesting a higher rate of recurrence to medications, hospitalizations, and specialist services in the CKD + sHPT patients with respect to CKD-only patients. These findings mirror those of the Spanish study, NEFRONA, that compared the differences in costs of pharmacological treatments and associated cardiovascular events between CKD patients with and without sHPT. Consistent with our data, the authors described substantially increased costs in both drug treatments and cardiovascular complications associated with sHPT, thus corroborating the view that a timely management during CKD progression could possibly lead to better clinical outcomes for the patients and reduced costs for healthcare systems [22]. In a different study, Schumock and colleagues reported that sHPT in pre-dialysis CKD patients is associated with significantly greater healthcare costs, inpatient hospitalizations, and a faster rate of disease progression than pre-dialysis CKD without sHPT [62]. However, it is also important to underline that a timely intervention with vitamin D therapy in CKD patients, poor vitamin D status, and sHPT have important implications in terms of both patients’ clinical outcomes and economic benefits. A recent cost-effectiveness analysis using the database of Medicare, the US government national health insurance, investigated the role of early vitamin D treatment in ND-CKD patients (stages 3–4) and focused on cardiovascular outcomes, fracture prevention, time to CKD stage 5/ESRD, mortality, quality-adjusted life years (QALYs). The study found that starting vitamin D treatment before CKD stage 5 (or even ESRD) resulted in cost savings of about USD 19.9 million [63].

The limitations of the present analysis are related to its observational nature; thus, the results must be interpreted based on data collection from administrative databases. Region/Local Health Units (LHUs) administrative databases have progressively improved the quality of the collected data. Nevertheless, some information may be missing, and when information was lacking for a given patient, that patient was excluded from the analysis. In addition, there was a lack of or limited clinical information on comorbidities and other potential confounders that could have influenced the present results. Since the comorbidities herein analyzed were addressed based on any available data before inclusion (using a proxy of diagnosis), there might be incomplete capture of these variables among patients. CKD patients were identified using the presence of a hospitalization discharge diagnosis, and some patients were not captured, thus underestimating the prevalence of CKD in our study cohort. The PSM methodology was designed using the variables assessed in the analysis to balance the two cohorts, thus the impact of other confounders, not retriable from the database, has not been investigated. Furthermore, the data on cardiovascular and fracture outcomes were not statistically significant as they were used as variables of the PSM, so the two populations were exactly comparable. In addition, primary care data could not be collected. However, our cohort of patients reflected real clinical practice by evaluating data from a subset of health-assisted individuals. 

## 5. Conclusions

The present analysis of real-world data allowed us to evaluate the epidemiology and clinical and economic impact of sHPT in CKD patients. The prevalence of specific CKD phenotypes, such as ND-CKD with and without sHPT, in our population was 50.6 cases per 100,000 people (0.05%) and 90.3 cases per 100,000 people (0.1%), respectively. The consistently lowered mortality rate found in CKD + sHPT patients treated with vitamin D, whatever the type, further underlines the usefulness of timely therapeutic interventions on sHPT, since the early phases of renal function decline. Given the broad spectrum of vitamin D pharmaceutical options, individual therapeutic approaches should be chosen based on the severity of renal and cardiovascular disease, as well as the profile of bone metabolism indices, with the final goal of controlling the morpho-functional changes elicited by sHPT itself. The presence of sHPT, also at an early CKD stage, was associated with a faster CKD progression to dialysis, increased mortality, and higher healthcare expenditures for patients’ management, suggesting that there is still an unmet need for better and early treatment of patients affected by sHPT.

## Figures and Tables

**Table 1:** Demographic and clinical characteristics at baseline of patients with a diagnosis of CKD without (CKD-only) and with sHPT diagnosis (CKD + sHPT), post PSM.

	CKD Only N = 1590	CKD + sHPT N = 1590	*p*-Value	Standardized Mean Difference
Age (mean, SD)	78.5 (12.5)	78.1 (12.1)	0.306	0.036
Male (n, %)	899 (56.5)	887 (55.8)	0.668	0.015
Diabetes (n, %)	661 (41.6)	695 (43.7)	0.223	0.043
Cardiovascular disease/treatments (n, %)	1558 (98.0)	1571 (98.8)	0.066	0.065
Hypertension (n, %)	1522 (95.7)	1565 (98.4)	<0.001	/
Dyslipidemia (n, %)	809 (50.9)	972 (61.1)	<0.001	/
Previous cardiovascular events (n, %)	773 (48.6)	786 (49.4)	0.645	/
Diuretics (n, %)	1225 (77.0)	1418 (89.2)	<0.001	/
Antiplatelets (n, %)	1092 (68.7)	1202 (75.6)	<0.001	/
Osteoporosis (n, %)	159 (10.0)	165 (10.4)	0.725	0.012
AVD (n, %)	0 (0.0)	738 (46.4)	<0.001	/
NVD (n, %)	0 (0.0)	432 (27.2)	<0.001	/
CKD Stage			<0.05	0.073
3 (n, %)	968 (60.9)	938 (59.0)		
4 (n, %)	514 (32.3)	499 (31.4)		
5 (n, %)	108 (6.8)	153 (9.6)		

**Table 2:** Baseline demographic and clinical characteristics of patients CKD + sHPT treated and untreated with AVD/NVD, post PSM.

	Untreated AVD/NVD N = 690	Treated AVD/NVD N = 690	*p*-Value	Standardized Mean Difference
Age (mean, SD)	76.2 (13.3)	76.3 (12.5)	0.836	0.011
Male (n, %)	425 (61.6)	412 (59.7)	0.474	0.039
Diabetes (n, %)	311 (45.1)	320 (46.4)	0.627	0.026
Cardiovascular disease/treatments (n, %)	680 (98.6)	681 (98.7)	0.817	0.012
Hypertension (n, %)	678 (98.3)	679 (98.4)	0.833	/
Dyslipidemia (n, %)	436 (63.2)	432 (62.6)	0.824	/
Previous cardiovascular events (n, %)	333 (48.3)	337 (48.8)	0.829	/
Diuretics (n, %)	610 (88.4)	608 (88.1)	0.867	/
Antiplatelets (n, %)	510 (73.9)	523 (75.8)	0.420	/
Osteoporosis (n, %)	34 (4.9)	51 (7.4)	0.057	0.103
CKD Stage			0.596	0.054
3 (n, %)	76.2 (13.3)	76.3 (12.5)		
4 (n, %)	425 (61.6)	412 (59.7)		
5 (n, %)	311 (45.1)	320 (46.4)		

## Data Availability

All data used for the current study are available upon reasonable request next to CliCon S.r.l., which is the body entitled for data treatment and analysis by Local Health Units.
